# The effects of alpha-lipoic acid supplementation on inflammatory markers among patients with metabolic syndrome and related disorders: a systematic review and meta-analysis of randomized controlled trials

**DOI:** 10.1186/s12986-018-0274-y

**Published:** 2018-06-05

**Authors:** Maryam Akbari, Vahidreza Ostadmohammadi, Reza Tabrizi, Moein Mobini, Kamran B. Lankarani, Mahmood Moosazadeh, Seyed Taghi Heydari, Maryam Chamani, Fariba Kolahdooz, Zatollah Asemi

**Affiliations:** 1 Health Policy Research Center, Institute of Health, Student Research Committee, Shiraz University of Medical Sciences, Shiraz, Iran; 2 Research Center for Biochemistry and Nutrition in Metabolic Diseases, Kashan University of Medical Sciences, Kashan, I.R Iran; 3 Kinesiology Department, University of Calgary, Calgary, AB Canada; 4 Health Policy Research Center, Institute of Health, Shiraz University of Medical Sciences, Shiraz, Iran; 5 Health Science Research Center, Addiction Institute, Mazandaran University of Medical Sciences, Sari, Iran; 6 Department of Gynecology and Obstetrics, School of Medicine, Iran University of Medical Sciences, Tehran, Iran; 7 Indigenous and Global Health Research, Department of Medicine, University of Alberta, Edmonton, Canada

**Keywords:** Alpha-lipoic acid, Inflammatory markers, Meta-analysis

## Abstract

**Objective:**

This systematic review and meta-analysis of randomized controlled trials (RCTs) was conducted to determine the effect of alpha-lipoic acid (ALA) supplementation on the inflammatory markers among patients with metabolic syndrome (MetS) and related disorders.

**Methods:**

We searched the following databases until November 2017: PubMed, MEDLINE, EMBASE, Web of Science, and Cochrane Central Register of Controlled Trials. Three reviewers independently assessed study eligibility, extracted data, and evaluated risk of bias of included primary studies. Statistical heterogeneity was assessed using Cochran’s Q test and I-square (I^2^) statistic. Data were pooled by using the random-effect model and standardized mean difference (SMD) was considered as the summary effect size.

**Results:**

Eighteen trials out of 912 potential citations were found to be eligible for our meta-analysis. The findings indicated that ALA supplementation significantly decreased *C-reactive protein* (CRP) (SMD = − 1.52; 95% CI, − 2.25, − 0.80; *P* < 0.001), *interlokin*-6 (IL-6) (SMD = − 1.96; 95% CI, − 2.60, − 1.32; *P* < 0.001), and tumor necrosis factor alpha levels (TNF-α) (SMD = − 2.62; 95% CI, − 3.70, − 1.55; *P* < 0.001) in patients diagnosed with metabolic diseases.

**Conclusion:**

In summary, the current meta-analysis demonstrated the promising impact of ALA administration on decreasing inflammatory markers such as CRP, IL-6 and TNF-α among patients with MetS and related disorders.

**Electronic supplementary material:**

The online version of this article (10.1186/s12986-018-0274-y) contains supplementary material, which is available to authorized users.

## Introduction

Increased pro-inflammatory markers and oxidative stress occurs in adipose tissues are the two factors that may play a key role in the incidence of metabolic-related comorbidities among patients with metabolic disorders [1]. Increased chronic inflammation is associated with increased risk of metabolic disorders, including type 2 diabetes mellitus (T2DM) [2] and arteriosclerosis, endothelial dysfunction, vascular calcification, increased activity of metalloproteinases, oxidative damage, and degradation of collagen [3–5]. It was reported that *metabolic syndrome (MetS*) is associated with a 2-fold increased risk of cardiovascular disease (CVD) over the next 5 to 10 years [6]. Inflammatory cytokines including interleukin-6 (IL-6) and tumor necrosis factor-alpha (TNF-α) are usually produced by different cells including endothelial, immune and arterial smooth muscle cells inducing the migration of additional immune cells into the atherosclerotic lesion and the activation of the acute phase C-reactive protein (CRP) in the liver [7, 8]. Increased levels of CRP are associated with increased risk of CVD and diabetes [9, 10]. In addition to CRP, other inflammatory biomarkers such as IL-6 and TNF-α may be correlated with the development of CVD in diabetic patients [11].

Complementary therapies such as antioxidants supplementation are recommended in patients with metabolic abnormalities to improve their nutritional status and boost their immune system [12]. Existing evidence has proved the beneficial effects of several antioxidants supplements including pentoxifylline [13] and lycopene [14] on reducing inflammation. Available data regarding the effects of alpha-lipoic acid (ALA) supplementation on inflammatory markers are controversial. In a study by Carbonelli et al. [15], obese Caucasian people showed significant reduction in CRP and TNF-α concentrations following ALA supplementation (800 mg/day) for 4 months. ALA supplementation (600–1000 mg/day) during a period ranging from 2 wk. to 1 year in patients with impaired glucose tolerance showed contradictory results. Zhang et al. [16] demonstrated that ALA supplementation decreased TNF-α and IL-6 while increased adiponectin levels, however others did not observe any beneficial effects of ALA on inflammatory markers [17, 18]. Discrepancies in these findings may be due to differences in study design, characteristics of study populations, dosage of ALA used and duration of the intervention.

We are aware of no systematic review or meta-analyses of randomized controlled trials (RCTs) evaluating the effect of ALA supplementation on inflammatory markers among patients with MetS and related disorders. Thus, the current meta-analysis was performed to summarize the available evidence regarding the effect of ALA supplementation on inflammatory markers among patients with MetS and related disorders.

## Materials and methods

### Search strategy and selection studies

We searched the following databases until November 2017: PubMed, MEDLINE, EMBASE, Web of Science, and Cochrane Central Register of Controlled Trials. Additionally, a manual search was conducted among the references lists of all eligible articles and review studies to identify potential articles that were not captured by the electronic searches. Three authors (VO, MM and MA) independently performed the literature search to retrieve RCTs that have examined the association between ALA supplementation and the inflammatory markers by using the following MeSH and text keywords: patients [“Mets” OR “disorders related to MetS” OR “diabetes” OR “T1DM” OR “T2DM” OR “overweight” OR “obese” OR “chronic kidney disease (CKD)” OR “hypertension” OR “high blood pressure” OR “dyslipidemia” OR “CVD”], intervention (“alpha-lipoic acid” OR “ALA” OR “α-lipoic acid” AND “supplementation” OR “intake”), and outcomes [“CRP” OR “IL-6” OR “TNF-α”]. Eligible studies were restricted to those RCTs published in English language.

### Inclusion and exclusion criteria

RCTs were selected using the following inclusion criteria: being a placebo-controlled randomized trial (either parallel or cross-over designs), human studies conducted in adults, the target population was patients diagnosed with metabolic diseases, and studies reported mean changes between pre- and post-intervention CRP and/or IL-6 and/or TNF-α following ALA supplementation for the intervention and placebos groups. Other types of human studies (cross-sectional, cohort studies), animal, in vitro studies, and review papers were excluded. Case reports or cases series, and the studies did not achieve the minimum quality assessment score, those receiving any non-steroidal anti-inflammatory drug or ALA supplements within the last month were also excluded from the study.

### Data extraction and quality assessment

Three authors (VO, MM, and MA) reviewed each trial and extracted all related data, independently. The disagreement among them was resolved by discussion with a fourth author (ZA). The quality of the included RCTs was assessed using the Cochrane Collaboration risk of bias tool based on the following information: randomization generation, allocation concealment, blinding of participants and outcome assessment, incomplete outcome data, and selective outcome reporting, as well as the other sources of bias. The extracted data included: first author, publication year, demographical variables, study design, sample size, dose of intervention, duration of study, type of intervention, type of disease, the mean and standard deviation (SD) for CRP, IL-6, and TNF-α.

### Data synthesis and statistical analysis

We preformed a comprehensive electronic and manual search to avoid publication bias. Additionally, Egger’s regression test was used to assess publication bias statistically [19]. Statistical heterogeneity was assessed using Cochran’s Q and I-square (I^2^) tests [20]. I^2^ greater than 50% or *P* < 0.05 was considered as significant heterogeneity. We estimated the difference between intervention (ALA supplementation) and placebo group by calculating the standardized mean difference (SMD) with 95% confidence interval (CI) using STATA software version 12.0 (Stata Corp., College Station, TX) and RevMan V.5.3 software (Cochrane Collaboration, Oxford, UK). Since the indications could effect on pooled SMD were different between included studies, we used random-effects models to perform meta-analyses. Subgroup and sensitivity analyses were conducted to assess the source of heterogeneity and to explore the contribution of each study to the reliability of the pooled mean difference, respectively. *P*-values < 0.05 were considered as statistically significant.

## Results

The process of the step by step study selection has shown in Additional file 1. Overall, 18 trials out of 912 potential citations were found to be eligible for this meta-analysis. Seven studies were RCTs design, and eleven were randomized, double-blind, placebo-controlled trials. Eleven trials have assessed the effects of ALA supplementation on CRP [21–31], eleven on IL-6 [16, 22–25, 30–35], and nine on TNF-α levels [16, 22, 24, 25, 28, 32, 33, 36, 37]. Intervention duration among included studies varied from 2 weeks to 12 months. The dosage of ALA supplements ranged from 300 to 600 (mg/day). Location of studies included; four studies in Italy [25, 28, 29, 33], three in Iran [23, 27, 37], three in China [16, 21, 22], two in Egypt [35, 36], one in Spain [30], one in Romania [32], two in United States [31, 34], one in Korea [26], and one in New Zealand [24]. Details of the included studies are summarized in Table 1. The quality of included trials is presented in Additional file 2.

**Table 1:** Characteristics of included studies

Authors (Ref)	Publication year	Sample size (control/intervention)	Country/population	Intervention (name and daily dose)	Duration	Presented data	Age (y) (control, intervention)	Results
Hong et al. [22]	2017	32/30	China/patients with diabetic nephropathy	450 mg ALA (IV) + 20 μg alprostadil	2 weeks	CRP, IL-6, TNF-α	65.82 ± 11.63, 67.24 ± 10.81	Decreased CRP, IL-6 and TNF-α
Sardu et al. [25]	2017	40/33	Italy/overweight patients with atrial fibrilation	600 mg ALA	12 months	CRP, IL-6, TNF-α	61.5 ± 8.1, 58.8 ± 6.7	Decreased CRP, IL-6 and TNF-α
Huerta et al. [30]	2016	21/19	Spain/overweight and obese women	300 mg ALA	10 weeks	CRP, IL-6	range: 20–50	Decreased CRP and IL-6
Huerta et al. [30]	2016	21/17	Spain/overweight and obese women	300 mg ALA + 1.3 g EPA	10 weeks	CRP, IL-6	range: 20–50	Decreased CRP
Marfella et al. [28]	2015	21/22	Italy/overweight patients with cardiomyopathy	600 mg ALA	12 months	CRP, TNF-α	63.9 ± 5.2, 63.7 ± 6.5	Decreased CRP and TNF-α
Safa et al. [37]	2014	31/30	Iran/patients with ESRD on hemodialysis	600 mg ALA	8 weeks	TNF-α	55.20 ± 13.43, 59.3 ± 10.47	No effect
Ahmadi et al. [23]	2013	24/20	Iran/hemodialysis patients	600 mg ALA	2 months	CRP, IL-6	48.9 ± 12.5, 48.8 ± 11.2	Decreased CRP and IL-6
Ahmadi et al. [23]	2013	24/24	Iran/hemodialysis patients	600 mg ALA + 400 IU vitamin E	2 months	CRP, IL-6	48.9 ± 12.5, 53.2 ± 9.8	Decreased CRP and IL-6
El-Nakib et al. [35]	2013	22/22	Egypt/patients with CRF on hemodialysis	600 mg ALA	3 months	IL-6	46.2 ± 14.4, 49.1 ± 16.2	No effect
Hegazy et al. [36]	2013	15/15	Egypt/patients with T1DM	600 mg ALA + insulin	4 months	TNF-α	11.1 ± 2.3, 11.9 ± 1.4	Decreased TNF-α
Cinteza al. [32]	2013	14/14	Romania/post acute stroke patients	600 mg ALA + other nutrients	2 weeks	IL-6, TNF-α	67.1 ± 10.85, 64 ± 10.85	Decreased IL-6 and TNF-α
Nasole et al. [33]	2013	6/10	Italy/patients with chronic leg wound and metabolic disease	600 mg ALA	2 weeks	IL-6, TNF-α	72,59	Decreased IL-6 and TNF-α
Nasole et al. [33]	2013	6/10	Italy/patients with chronic leg wound and metabolic disease	600 mg R-(+)-lipoic acid (RLA)	2 weeks	IL-6, TNF-α	72,72	Decreased IL-6 and TNF-α
Khabbazi et al. [27]	2012	28/24	Iran/patients with ESRD on hemodialysis	600 mg ALA	8 weeks	CRP	54.04 ± 13.96, 53.83 ± 13.29	Decreased CRP
Manning et al. [24]	2012	39/34	New Zealand/patients with MetS	600 mg ALA	12 months	CRP, IL-6, TNF-α	57 ± 9, 55 ± 10	No effect
Zhang et al. [16]	2011	9/13	China/obese patients with impaired glucose tolerance	600 mg ALA (IV)	2 weeks	IL-6, TNF-α	52.6 ± 6.2, 52.5 ± 8.2	Decreased IL-6 and TNF-α
Xiang et al. [21]	2011	30/30	China/patients with impaired fasting glucose	600 mg ALA (IV)	3 weeks	CRP	58 ± 9, 58 ± 10	Decreased CRP
Gianturco et al. [29]	2009	7/7	Italy/patients with NIDDM	400 mg ALA	4 weeks	CRP	58 ± 16, 61 ± 7	No effect
Chang et al. [26]	2007	25/25	Korea/diabetic ESRD patients on hemodialysis	600 mg ALA	12 weeks	CRP	66 ± 7, 63 ± 6	No effect
Sola et al. [34]	2005	14/15	USA/patients with MetS	300 mg ALA	4 weeks	IL-6	44 ± 13, 46 ± 15	Decreased IL-6
Sola et al. [34]	2005	14/15	USA/patients with MetS	300 mg ALA + 150 mg irbesartan	4 weeks	IL-6	44 ± 13, 48 ± 12	Decreased IL-6
Romos et al. [31]	2012	28/30	USA/patients with CKD	600 mg ALA + 666 IU tocopherols	8 weeks	CRP, IL-6	64.5 ± 8.8, 58.6 ± 12.0	Decreased IL-6

*ALA* alpha-lipoic acid, *CRF* chronic renal failure, *CKD* chronic kidney disease, *ESRD* end-stage renal disease, *IV* intravascular, *IL-6* interlokin-6, *CRP* C-reactive protein, *MetS* metabolic syndrome, *NIDDM* non-insulin-dependent diabetes mellitus, *TNF-α* tumor necrosis factor alpha, *T1DM* type 1 diabetes mellitus, *T2DM* type 2 diabetes mellitus

### Main outcomes

**Fig. 1:**
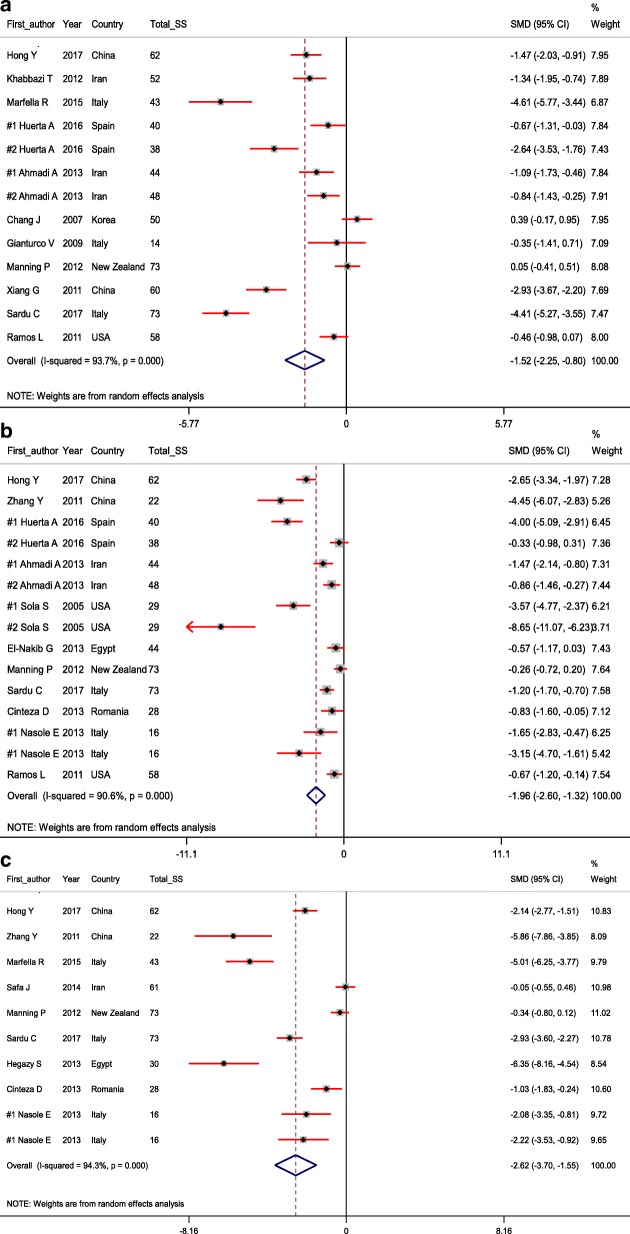
**a**-**c** Meta-analysis glycemic control standardized mean differences estimates for (**a**) high-sensitivity C-reactive protein, (**b**) for interlokin-6, and (**c**) for tumor necrosis factor alpha in alpha-lipoic acid supplements and placebo groups (CI = 95%)

The results of current meta-analysis showed that ALA supplementation significantly decreased CRP (SMD = − 1.52; 95% CI, − 2.25, − 0.80; *P* < 0.001; I^2^: 93.7), IL-6 (SMD = − 1.96; 95% CI, − 2.60, − 1.32; *P* < 0.001; I^2^: 90.6), and TNF-α levels (SMD = − 2.62; 95% CI, − 3.70, − 1.55; *P* < 0.001; I^2^: 94.3) in patients with MetS and related disorders (Table 2 and Fig. 1).

**Table 2:** Estimation of the standardized difference means of related indictors with CI 95% between the intervention and placebo groups

Variables		Number of study	Standardized mean difference	CI 95%	Heterogeneity		
					I^2^ (%)	Q	*P*-value
CRP	Intervention group (after vs. before)	11	−0.88	−1.55, − 0.21	92.4	130.85	< 0.001
	Placebo group (after vs. before)	11	−0.29	− 0.67, 0.09	80.3	50.87	< 0.001
	Change intervention group vs. placebo group	13	−1.52	−2.25, −0.80	93.7	191.17	< 0.001
IL-6	Intervention group (after vs. before)	13	−0.99	−1.48, − 0.51	85.0	79.92	< 0.001
	Placebo group (after vs. before)	13	0.03	−0.16, 0.22	17.8	14.59	0.264
	Change intervention group vs. placebo group	15	−1.96	−2.60, − 1.32	90.6	149.58	< 0.001
TNF-α	Intervention group (after vs. before)	10	−1.41	−2.03, −0.79	87.0	69.13	< 0.001
	Placebo group (after vs. before)	10	−0.33	− 0.72, 0.05	71.4	31.50	< 0.001
	Change intervention group vs. placebo group	10	−2.62	−3.70, −1.55	94.3	157.51	< 0.001

*IL-6* interlokin-6, *CRP* C-reactive protein, *TNF-α* tumor necrosis factor alpha

We also performed subgroup analyses according to geographic area (Asia, European, USA, Oceania, and Africa), dosage of ALA supplements (> 600 vs. ≤600 mg/day), co-administration with other nutrients (ALA vs. ALA plus other nutrients), duration of the intervention (≥8 vs. < 8 weeks), and type of diseases (diabetic, ESRD vs. other diseases). We found that heterogeneity may decrease by duration of the intervention (< 8 weeks = I^2^: 89.9 and ≥ 8 weeks = I^2^:85.1 vs. overall I^2^:90.6%) and type of diseases (diabetic = I^2^: 75.1 and other = I^2^:89.5 vs. overall I^2^:90.6%) for IL-6 and type of diseases (diabetic = I^2^: 92.8 and other = I^2^:94.0 vs. overall I^2^:94.3%) for TNF-α levels (< 8 weeks = I^2^: 88.2 and ≥ 8 weeks = I^2^:92.1 vs. overall I^2^:94.5%). The detailed of subgroup analysis are presented in Table 3.

**Table 3:** The assess of association between alpha-lipoic acid supplementation on inflammatory markers based on subgroup analysis

Variables		Number of SMD included	Subgroups	Pooled OR (random effect)	95% CI	I^2^ (%)	overall I^2^(%)
CRP	Geographic area	6	Asia	−1.20	−2.02, − 0.38	90.8	93.7
		5	European	−2.52	−4.26, −0.78	94.8	
		1	USA	−0.46	− 0.98, 0.07	–	
		1	Oceania	0.05	−0.41, 0.51	–	
	Dosage of ALA (mg/day)	4	< 600	−1.29	−2.16, −0.42	81.4	
		9	≥600	−1.64	−2.61, −0.66	95.4	
	Type of intervention	9	ALA	−1.63	−2.67, −0.58	95.3	
		4	ALA plus other nutrients	−1.30	−2.10, −0.50	85.0	
	Duration of study (week)	10	≥8	−1.50	−2.35, −0.65	94.4	
		3	< 8	−1.63	−2.93, −0.32	88.6	
	Type of diseases	3	Diabetic	−1.63	−2.93, −0.32	88.6	
		10	Other	−1.50	−2.35, −0.65	94.4	
IL-6	Geographic area	4	Asia	−2.18	−3.34, −1.02	88.7	90.6
		6	European	−1.75	−2.71, −0.78	87.4	
		3	USA	−4.10	−7.68, −0.52	96.3	
		1	Oceania	−0.26	−0.72, 0.20	–	
		1	Africa	−0.57	−1.17, 0.03	–	
	Dosage of ALA (mg/day)	5	< 600	−3.59	−5.50, −1.68	94.8	
		10	≥600	−1.22	− 1.70, −0.74	78.4	
	Type of intervention	8	ALA	−2.00	−2.88, −1.11	90.9	
		7	ALA plus other nutrients	−1.98	−3.01, −0.95	91.7	
	Duration of study (week)	8	≥8	−1.07	− 1.62, −0.52	85.1	
		7	< 8	−3.30	−4.61, −1.99	89.9	
	Type of diseases	2	Diabetic	−3.40	−5.13, −1.66	75.1	
		13	Other	−1.72	−2.35, − 1.09	89.5	
TNF-α	Geographic area	3	Asia	−2.44	−4.67, −0.21	95.9	94.3
		5	European	−2.63	−3.87, −1.39	87.2	
		1	Oceania	−0.34	− 0.80, 0.12	–	
		1	Africa	−6.35	−8.16, −4.54	–	
	Dosage of ALA (mg/day)	1	< 600	−2.14	−2.77, −1.51	–	
		9	≥600	−2.71	−3.94, − 1.48	94.7	
	Type of intervention	6	ALA	−2.56	−4.08, −1.03	95.8	
		4	ALA plus other nutrients	−2.73	−4.26, −1.20	89.3	
	Duration of study (week)	5	≥8	−2.78	−4.57, −0.99	96.8	
		5	< 8	−2.40	−3.46, −1.33	80.2	
	Type of diseases	3	Diabetic	−4.68	−7.82, −1.55	92.8	
		7	Other	−1.89	−3.04, −0.73	94.0	

*ESRD* end-stage renal disease, *IL-6* interlokin-6, *CRP* C-reactive protein, *TNF-α* tumor necrosis factor alpha

In sensitivity analysis, we found no significant difference between the pre- and post-sensitivity analysis for all inflammatory markers. The smallest and greatest pooled SMDs in the sensitivity analyses for the level of inflammatory markers are shown in Additional file 3. Egger’s regression tests showed no significant publication bias for the effects of ALA on CRP (B = − 11.35, *P* = 0.01). We found publication bias for IL-6 (B = − 6.88, *P* = 0.00) and TNF-α (B = − 7.28, P = 0.01), so we non parametric method was applied (Duval and Tweedie) to estimate the findings of censored studies. Findings showed that the summary of effect size for IL-6 and TNF-α did not significantly changed between before and after inclusion of censored studies for CRP (SMD = − 1.69; 95% CI, − 2.48, − 0.90), IL-6 (SMD = − 1.96; 95% CI, − 2.60, − 1.32), and TNF-α (SMD = − 2.62; 95% CI, − 3.70, − 1.55).

## Discussion

This systematic review and meta-analysis assessed the effect of ALA supplementation on inflammatory markers in patients with MetS and related disorders. Our findings supported the beneficial impact of ALA administration on lowering inflammatory markers in patients suffering from metabolic syndrome and related disorders.

Few studies have reported the beneficial effects of antioxidant supplementation on inflammatory cytokines. In a meta-analysis conducted by Ju et al. [38], selenium supplementation significantly decreased serum CRP levels in patients with coronary heart disease, suggesting its potential impact on reducing inflammation in chronic conditions. In addition, supplementation with vitamin E in the form of either α-tocopherol or γ-tocopherol resulted in a significant reduction in CRP concentrations [39]. Available information regarding the effects of ALA supplementation on inflammatory cytokines is inconclusive. ALA supplementation for 12 months significantly decreased serum levels of common markers of inflammation in ablated patients [25]. Furthermore, dietary supplementation with ALA for 10 weeks significantly improved systemic inflammation and cardiovascular disease-related risk factors in healthy overweight women [30]. However, no benefits of resveratrol supplementation were reported on cardiovascular risk factors in the meta-analysis conducted by Sahebkar et al. [40]. In another study, taking ALA supplements for 8 weeks did not affect IL-8 and TNF-α levels in hemodialysis patients [37]. Increased inflammatory markers, especially TNF-α, might promote insulin resistance, and alter expression of cytokines in adipose tissues which is considered an important link between *MetS* and insulin resistance [41]. In addition, high levels of inflammatory markers in diabetic patients and those suffering from diabetic nephropathy are positively correlated with the severity of albuminuria [42]. Local inflammation plays also an important role in the development of diabetic retinopathy [43].

ALA intake may reduce inflammatory markers through scavenging free radicals, down-regulating pro-inflammatory redox-sensitive signal transduction processes including nuclear factor kappa B translocation, leading to lower release of other free radicals and cytotoxic cytokines [44, 45]. Moreover, ALA administration improves cellular antioxidant capacity and phases 2 enzymes such as catalase, reduced glutathione, glutathione reductase, and glutathione-S-transferase [46]. ALA can also inhibit the activation of serine kinases including IKKβ to suppress inflammatory cytokines [47]. Zhang et al. [48] mentioned to ALA potential to inhibit TNF-α-induced I kappa B kinase activation. It is speculated that the ALA treatment effects might be influenced by its baseline values and improved blood levels over time. In the current meta-analysis it was not possible to consider the effect of baseline ALA values in determining the impact of it on inflammatory markers. Furthermore, different geographical latitudes where study conducted might further complicate the effect of baseline ALA values. Overall, on top of those explained above, different study designs, sample size, different dosages of ALA used along with characteristics of study participants might explain the discrepancies among different studies.

There are several strengths for this study. Higher numbers of studies included in this analysis and longer period of supplementation in included trials have added to the value of this meta-analysis. All included studies were placebo-controlled randomized trials with acceptable methodological quality and the least probable chance of bias. Further, we relied on independent judgment in which different reviewers independently performed the systematic review process.

The current meta-analysis had a few limitations. There were few eligible RCTs, and most of them had a modest number of participants. Various doses of ALA were administered for intervention in the included studies. We were unable to evaluate the dose response association between supplementation dose and inflammatory markers due to the low number of studies included. In addition, we did not evaluate the residual confounding and bias of each study that could not be addressed through pooling. Considerable heterogeneity across studies made our findings complicated to interpret the main outcomes. Thus, evaluation of heterogeneity is a crucial part of any meta-analysis.

## Conclusions

Overall, the current meta-analysis supported the beneficial impacts of ALA administration on decreasing inflammatory markers such as CRP, IL-6 and TNF-α among patients with MetS and related disorders.

## Additional files

Additional file 1: Literature search and review flowchart for selection of studies. (DOC 44 kb)

Additional file 2: The methodological quality of included studies (risk of bias). (DOC 44 kb)

Additional file 3: The effects of alpha-lipoic acid supplementation on inflammatory markers based on sensitivity analysis. (DOC 33 kb)

## Abbreviations

ALA: Alpha-lipoic acid
CRF: Chronic renal failure
CRP: C-reactive protein
ESRD: End-stage renal disease
IL-6: Interlokin-6
IV: Intravascular
MetS: Metabolic syndrome
NIDDM: Non-insulin-dependent diabetes mellitus
T1DM: Type 1 diabetes mellitus
T2DM: Type 2 diabetes mellitus
TNF-α: Tumor necrosis factor alpha
