# Supplementary material for: The effects of alpha-lipoic acid supplementation on inflammatory markers among patients with metabolic syndrome and related disorders: a systematic review and meta-analysis of randomized controlled trials
Source: Nutr Metab (Lond). 2018 Jun 5;15:39. doi: 10.1186/s12986-018-0274-y (PMC5989440; doi:10.1186/s12986-018-0274-y)
Supplement: Supplementary file 1 — Literature search and review flowchart for selection of studies. (DOC 44 kb) [file 12986_2018_274_MOESM1_ESM.doc]

Articles screened by title and abstract (n=197)

Full text articles assessed for eligibility (n=48)

Studies included in this study (n=18)

1. Alpha-lipoic acid (n=14)

2. Alpha-lipoic acid plus other nutrients (n=4)

Article excluded (n=715) due to duplicate articles, not randomized controlled trials, review and not human

Excluded non-relevant articles (n=149)

Articles excluded (n=30):

1. Not metabolic disease (n=16)

2. Data presentation inappropriate for meta-analysis (n=12)

3. Not placebo (n=2)

Articles identified through electronic database search (n=912)

**Additional file 1** Literature search and review flowchart for selection of studies
