# Supplementary figures and images for: The effects of alpha-lipoic acid supplementation on inflammatory markers among patients with metabolic syndrome and related disorders: a systematic review and meta-analysis of randomized controlled trials
Source: Nutr Metab (Lond). 2018 Jun 5;15:39. doi: 10.1186/s12986-018-0274-y (PMC5989440; doi:10.1186/s12986-018-0274-y)

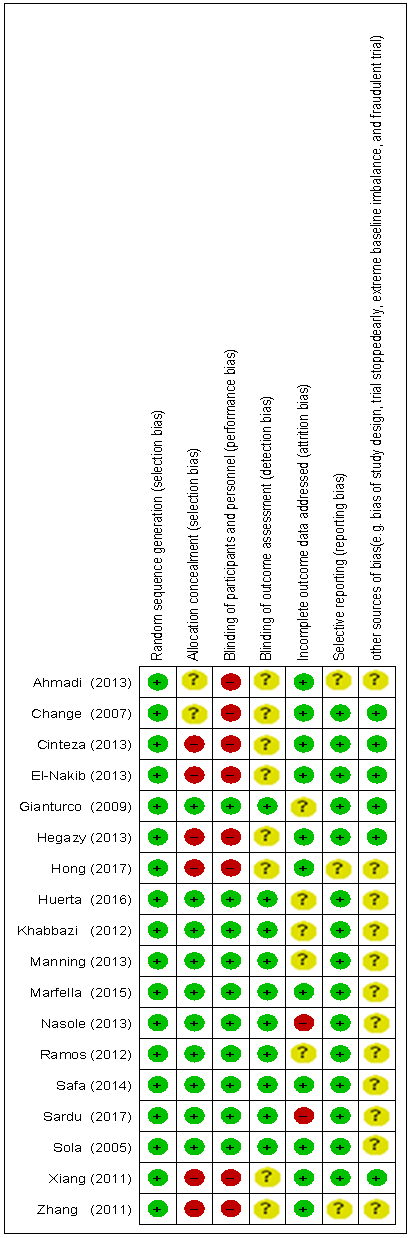


**Additional file 2** The methodological quality of included studies (risk of bias)

Supplement: Supplementary file 2 — The methodological quality of included studies (risk of bias). (DOC 44 kb) [file 12986_2018_274_MOESM2_ESM.doc]
