# Supplementary material for: The effects of alpha-lipoic acid supplementation on inflammatory markers among patients with metabolic syndrome and related disorders: a systematic review and meta-analysis of randomized controlled trials
Source: Nutr Metab (Lond). 2018 Jun 5;15:39. doi: 10.1186/s12986-018-0274-y (PMC5989440; doi:10.1186/s12986-018-0274-y)
Supplement: Supplementary file 3 — The effects of alpha-lipoic acid supplementation on inflammatory markers based on sensitivity analysis. (DOC 33 kb) [file 12986_2018_274_MOESM3_ESM.doc]

**Additional file 3** The effects of alpha-lipoic acid supplementation on inflammatory markers based on sensitivity analysis

| Variables | Pre-sensitivity analysis | | | Upper & lower of effect size | Post-sensitivity analysis | | |
| --- | --- | --- | --- | --- | --- | --- | --- |
|  | No. of studies included | Pooled SMD  (random effect) | 95% CI |  | Pooled SMD  (random effect) | 95% CI | Excluded  studies |
| CRP | 13 | -1.52 | -2.25, -0.80 | Upper | -1.27 | -1.27, -1.91 | Sardu |
|  |  |  |  | Lower | -1.68 | -2.42, -0.94 | Chang |
| IL-6 | 15 | -1.96 | -2.60, -1.32 | Upper | -1.66 | -2.24, -1.09 | 2 Sola |
|  |  |  |  | Lower | -2.10 | -2.78, -1.43 | Manning |
| TNF-α | 10 | -2.62 | -3.70, -1.55 | Upper | -2.25 | -3.29, -1.21 | Hegazy |
|  |  |  |  | Lower | -2.94 | -4.09, -1.79 | Safa |

IL-6, interlokin-6; CRP, C-reactive protein; TNF-α, tumor necrosis factor alpha.
